# How are MCPIP1 and cytokines mutually regulated in cancer-related immunity?

**DOI:** 10.1007/s13238-020-00739-1

**Published:** 2020-06-16

**Authors:** Ruyi Xu, Yi Li, Yang Liu, Jianwei Qu, Wen Cao, Enfan Zhang, Jingsong He, Zhen Cai

**Affiliations:** 1 Bone Marrow Transplantation Center, The First Affiliated Hospital, School of Medicine, Zhejiang University, Hangzhou, 310006 China; 2 Institution of Hematology, Zhejiang University, Hangzhou, 310006 China

**Keywords:** MCPIP1, cytokines, cancer-related immunity, RNase, deubiquitinase

## Abstract

Cytokines are secreted by various cell types and act as critical mediators in many physiological processes, including immune response and tumor progression. Cytokines production is precisely and timely regulated by multiple mechanisms at different levels, ranging from transcriptional to post-transcriptional and posttranslational processes. Monocyte chemoattractant protein-1 induced protein 1 (MCPIP1), a potent immunosuppressive protein, was first described as a transcription factor in monocytes treated with monocyte chemoattractant protein-1 (MCP-1) and subsequently found to possess intrinsic RNase and deubiquitinase activities. MCPIP1 tightly regulates cytokines expression via various functions. Furthermore, cytokines such as interleukin 1 beta (IL-1B) and MCP-1 and inflammatory cytokines inducer lipopolysaccharide (LPS) strongly induce MCPIP1 expression. Mutually regulated MCPIP1 and cytokines form a complicated network in the tumor environment. In this review, we summarize how MCPIP1 and cytokines reciprocally interact and elucidate the effect of the network formed by these components in cancer-related immunity with aim of exploring potential clinical benefits of their mutual regulation.

## INTRODUCTION

The idea that inflammation contributes to the onset of cancer, which is now well accepted and considered a vital characteristic of cancer, can be traced back to the 19th century (Coussens et al., 2013; Elinav et al., 2013; Coffelt and De Visser, 2014; Maman and Witz, 2018). According to extensive research results over the past decade, approximately 25% of human cancers are caused by chronic inflammation (Mantovani et al., 2008). In contrast to self-limiting innate inflammation, which acts as a defense for the fight against invading pathogens, persistent and dysregulated chronic inflammation increases the risk of human cancer. As chronic inflammation is related to sustained and continuous tissue damage and repair, it leads to the chaotic proliferation of cells, resulting in accumulation of atypical cell populations and even neoplasia (Prach et al., 1997; Houghton et al., 2004).

The importance of intracellular communication between malignant cells and immune cells within the tumor microenvironment has long been recognized (Stoeltzing et al., 2006). Under stimuli, host immune cells secrete cytokines and other small inflammatory proteins to fight against tumors, but these released cytokines sometimes conversely activate malignant cells, causing specific mutations and epigenetic changes in cancer cells (Galdiero et al., 2018). Correspondingly, highly proliferative cancer cells will produce increased levels of cytokines, attracting immune cells and regulating the gene expression pattern of host cells (Chitu and Stanley, 2006; Colotta et al., 2009).

Cytokines are an indispensable component of the intracellular feedback loop between tumor and host immunity (Diakos et al., 2014). They regulate cancer progression through many mechanisms, including acceleration of the epithelial-to-mesenchymal transition, stimulation of angiogenesis and augmentation of metastasis (Fiori et al., 2019). More specifically, cytokines such as IL-1B, interleukin 6 (IL-6), interferon gamma (IFN-γ) and interleukin 10 (IL-10) can effectively activate immunosuppressive pathways in myeloid-derived suppressive cells (MDSCs) and induce the differentiation of MDSCs into tumor-protective dendritic and macrophage cells (Dysthe and Parihar, 2020). Chemokines, a kind of chemotactic cytokines, also profoundly contribute to promoting tumor progression. Derived from various cell types, chemokines play a key role in the metastatic spread of tumor and are usually overexpressed in highly invasive tumors (Marcuzzi et al., 2019).

In addition to direct regulation by transcription factors, such as NF-κB and STAT3, the regulation of cytokines production by post-transcriptional mechanisms has been well studied (Karin and Greten, 2005; Takeuchi, 2018). Timely and precisely adjusting cytoplasmic concentrations of cytokines mRNA towards different status is important for immunity homeostasis. Although transcription is the first step, many post-transcriptional processes such as mRNA splicing, degradation, polyadenylation and translation are involved in the tight regulation of cytokines (Fu and Blackshear, 2017). For example, restricted TNF production is related to the adenylate-uridylate-rich elements (AREs)-mediated and constitutive decay element (CDE)-mediated decay of *TNF* mRNA (Fu and Blackshear, 2017). Strictly regulation of mRNA turnover kinetics is essential for cells to adjust their translation potential due to diverse stimuli. After cells are activated, the half-lives of the mRNAs of many cytokines are significantly shortened because of mRNA decay (Fu and Blackshear, 2017), in which the newly recognized immunosuppressive protein MCPIP1 plays an important role (Matsushita et al., 2009).

**Figure 1:**
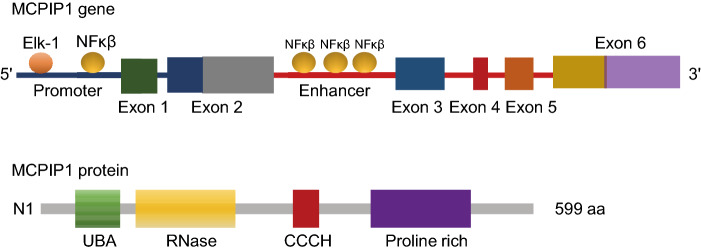
**Schematic structures of human MCPIP1 gene and protein**. The binding sites of transcription factors Elk-1 and NF-κβ are showed in *MCPIP1* gene. The location of promoter, Enhancer and Exons are also indicated in *MCPIP1* gene. The protein domains are presented. UBA: ubiquitin-associated domain; RNase: ribonuclease domain

MCPIP1, also known as Regnase-1 and ZC3H12A, a 65.8-kDa member of the CCCH zinc finger proteins that contains a PilT N-terminus (PIN) domain-like RNase domain and a ubiquitin (Ub)-associated domain at its N-terminus, can act as a regulator of RNA metabolic processes (Xu et al., 2012) (Fig. 1). It recognizes stem-loop structures with specific pyrimidine-purine-pyrimidine loop sequences in the 3′ untranslated regions (3′UTRs) of mRNA and then degrades those transcripts. In addition to directly destabilizing mRNAs by its intrinsic RNase activity, MCPIP1 can act as a deubiquitinating enzyme to regulate protein expression (Liang et al., 2010). Research results showed that MCPIP1 deubiquitinates TRAF6 by forming a complex with USP10 and TANK. As CCCH zinc finger proteins usually shuttle between cellular compartments, MCPIP1 was first found in the nucleus when MCPIP1-GFP was expressed in HEK293 cells (Mino et al., 2015), but some studies then identified MCPIP1 on ribosomes on the endoplasmic reticulum, and some studies indicated that MCPIP1 could form granule-like structures in the cytoplasm that interact with the miRISC to regulate miRNA effector pathways (Huang et al., 2015).

In this review, we focus on how MCPIP1 regulates inflammatory cytokines expression in the cancer microenvironment though its various functional domains and identify a feedback loop between MCPIP1 and cytokines, exploring feasible strategies to disturb or reinforce their mutual effects to benefit clinical practice.

## HOW DOES MCPIP1 REGULATE CYTOKINES PRODUCTION?

**Figure 2:**
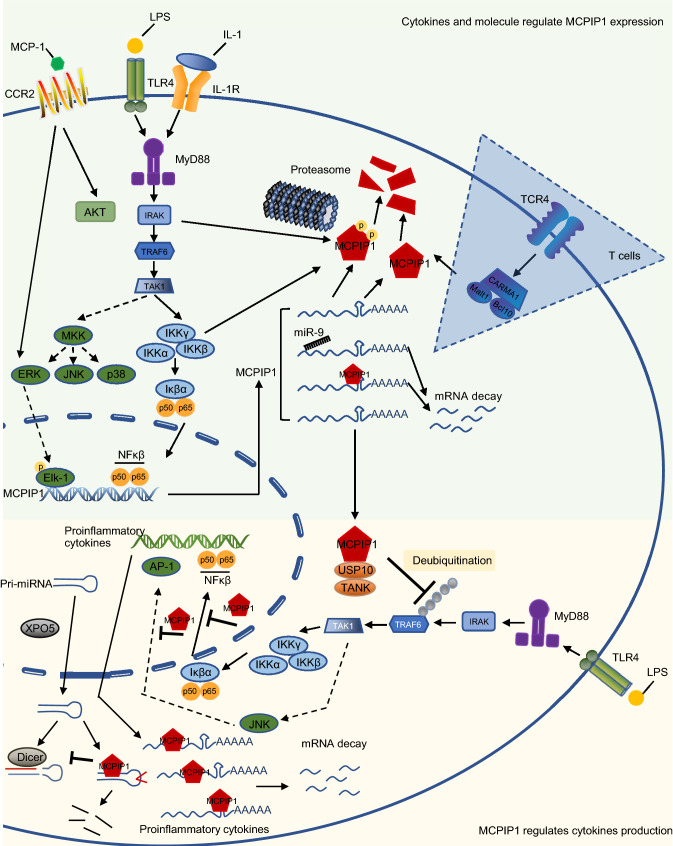
**The mechanism how MCPIP1 and cytokines mutually regulated in macrophages**. The upper green part indicates the mechanism how cytokines and LPS regulate MCPIP1 expression. Activation of TLR4 by LPS, or IL-1R by IL-1, subsequently activates the inhibitor of NF-κβ kinase (IKK) complex, leading to the phosphorylation and degradation of IκBα. Then NF-κβ is released and translocate to the nucleus to induce the transcription of *MCPIP1* gene. MAPkinase pathway is also activated and phosphorylated Elk-1 promotes *MCPIP1* transcription. Post-transcriptional regulation happened in cytoplasm, *MCPIP1* mRNAs interact with miR-9 or MCPIP1 protein and undergo degradation. Those translated MCPIP1 protein can be phosphorylated by NF-κβ signaling and then degraded by proteasome machinery. The blue triangle indicates a different mechanism of MCPIP1 protein degradation in T cells. It’s mediated by protease Malt1. The Lower yellow part indicates the mechanism how MCPIP1 protein regulates cytokines production. MCPIP1 interacts with USP10 and TANK to inhibit NF-κβ signaling by deubiquitinating the activated TRAF6. In cytoplasm, MCPIP1 interacts with cytokines transcripts to induce mRNA decay. In addition, its anti-Dicer RNase activity inhibits miRNAs maturation. In nucleus, MCPIP1 directly inhibits translocated NF-κβ and AP-1 bind to the target proinflammatory cytokines genes, then suppresses proinflammatory cytokines transcription

Great effort has been made to elucidate the mutual regulation between MCPIP1 and cytokines (Xu et al., 2012). In the following section, we discuss four common mechanisms by which MCPIP1 tightly regulates cytokines production and maintains immune homeostasis. We have summarized this mutual regulatory network in Fig. 2. Considering cytokines and MCPIP1 may act differently in human and murine system, we also summarized experimental models used in studies in Table 1, which suggests the interplay between cytokines and MCPIP1 is similar in both species.

**Table 1:** The interplay between cytokines and MCPIP1

	Substrates/Regulators	Models	Effect
**MCPIP1 regulates cytokines production**	TNFα	Mouse macrophage cell line RAW264.7 (Liang J et al., 2008) MCPIP1^−/−^ mice (Huang S et al., 2013) Mouse Embryonic Fibroblasts (MEF) (Niu J et al., 2013) MCPIP1 myelo-KO mice peritoneal macrophages (Kapoor N et al. 2015) Human HepG2 cell line (Skalniak L et al., 2009)	Inhibits p65-induced promoter activation of *TNF* in LPS treated cells; Inhibits LPS-activated JNK signaling Prevents NF-κΒ activation by removing the ubiquitins from TRAF6; Suppresses the synthesis of miR155 and miR125
	IL-6	MCPIP1^−/−^ mice macrophage (Matsushita K et al., 2009) Mouse stromal cell line ST-2 (Garg A et al., 2015) Mouse macrophage cell line RAW264.7 (Mino T et al., 2015) Mouse Embryonic Fibroblasts (MEF) (Niu J et al., 2013) (Mino T et al., 2015) Human HeLa cell line (Mino T et al., 2015) Human HEK293 cell line (Mino T et al., 2015)	Destabilizes *IL6* mRNA; Inhibits NF-κΒ activation
	IL-12	MCPIP1^−/−^ mice macrophage (Matsushita K et al., 2009)	Destabilizes *IL12p40* mRNA
	MCP-1	MCPIP1 myelo -KO mice peritoneal macrophages (Kapoor N et al., 2015) Mouse macrophage cell line RAW264.7 (Liang J et al., 2008)	Suppresses the synthesis of miR155 and miR125; Inhibits NF-κΒ activation
	IL-2	Mouse CD4^+^ T lymphocytes (Li M et al., 2012) Human blood CD4^+^ T lymphocytes (Li M et al., 2012)	Destabilizes *IL2* mRNA
	IL-1B	Human skin fibroblasts (Mizgalska D et al., 2009)	Destabilizes mRNA
	IL-8	Human HeLa cells (Dobosz E et al., 2016) Human Caco-2 cell line (Dobosz E et al., 2016)	Destabilizes *IL8* mRNA
	IL-4	MCPIP1^−/−^ mice spleen cells (Miao R et al., 2013) Human Jurkat T cells (Suzuki H et al., 2011)	Destabilizes *IL4* mRNA Suppresses the synthesis of miR155;
**Cytokines and molecules regulate MCPIP1 expression**	TNFα	Human THP-1 cells (Liang J et al., 2008) Human umbilical vein endothelial cells (HUVECs) (Qi Y et al., 2010) Human U937 cell line (Mizgalska D et al., 2009)	Induces *MCPIP1* expression
	MCP-1	Mouse macrophage cell line RAW264.7 (Zhou L et al., 2006) Human peripheral blood monocytes (Zhou L et al., 2006) Human HUVECs (Niu J et al., 2008)	Induces Elk-1 phosphorylation Activates Akt signaling
	IL-4	Mouse peritoneal macrophages (Kapoor N et al., 2015)	Induces KLF4 to activate *MCPIP1* transcription
	IL-1B	MCPIP1^−/−^ mice peritoneal macrophages and MEFs (Iwasaki H et al., 2011) Human THP-1 cells (Liang J et al., 2008) Human HepG2 cell line (Skalniak L et al., 2009) (Mizgalska D et al., 2009)	Activates NF-κB signaling and ERK MAPkinase pathway to induce *MCPIP1* expression; Activates IRAK1 and IKKβ to phosphorylate MCPIP1 protein which undergoes degradation by ubiquitin proteasome; Upregulates MCPIP1 protein to destabilize *MCPIP1* mRNA itself
	IL-1α	Human synovial fibroblasts from a patient with osteoarthritis (Dhamija S et al., 2013)	Increases ribosome occupancy of *MCPIP1* mRNA
	IL-17	Mouse stromal cell line ST-2 (Garg A et al., 2015) Primary mouse embryo fibroblasts (Sønder S et al., 2011) Human HeLa cells (Dhamija S et al., 2013) Human oral keratinocytes (Garg A et al., 2015)	Stabilizes *MCPIP1* mRNA; Recruits CIKS/Act1 to activate NK-κB and then induce *MCPIP1* expression
	LPS	Mouse RAW264.7 (Liang J et al., 2008); (Huang S et al., 2013) (Dhamija S et al., 2013) Mouse bone marrow-derived macrophages (Liang J et al., 2008) Rat microglial cells (Yao H et al., 2014) MCPIP1^−/−^ mice peritoneal macrophages and MEFs (Iwasaki H et al., 2011) Human THP-1 derived macrophages (Liang J et al., 2008) Human peripheral blood mononuclear cells (Dhamija S et al., 2013)	Activates NF-κB signaling and ERK MAPkinase pathway Inhibits miR-9 to stabilize *MCPIP1* mRNA Activates IRAK1 and IKKβ to phosphorylate MCPIP1 protein which undergoes degradation by ubiquitin proteasome

### At the transcriptional level

MCPIP1 was first described as a transcription factor in human monocytes after treatment with MCP-1(Zhou et al., 2006). The transcription factor-like activity of MCPIP1 was confirmed by luciferase reporter system. HEK293 cells cotransfected with GAL4-MCPIP1 and pGal4-Luc reporter showed MCPIP1 activated luciferase reporter gene transcription to 865-fold/mg protein and mutation of the CCCH zinc finger domain or proline-rich domain was found to drastically affect its transcriptional activity. It is determined that the transcription factor-like activity of MCPIP1 contributes to apoptotic genes transcription and leads to cell death. Another study showed that MCPIP1 can act as a transcription factor by CHIP analysis, with the results indicating that *Cadherin* 12 (*CDH12*) and *CDH19* are targets of MCPIP1 (Niu et al., 2008). MCPIP1 transcriptionally activates the expression of these angiogenesis-related genes, thus promoting capillary-like tube formation.

In addition, MCPIP1 regulates genes transcription by interacting with other potent transcription factors. LPS-induced TLR signaling activation is critical for immune defense to infection. Stimulation of macrophages by LPS strongly activates multiple signaling pathways to rapidly release proinflammatory cytokines, such as TNFα, IL-1B, IL-6, IL-12, and IL-18 (Wynn et al., 2013). The NF-κB pathway plays an important role in regulating proinflammatory cytokines expression. The results from Liang et al. showed that MCPIP1, unlike other CCCH zinc finger proteins, does not regulate *TNF* or *MCP-1* mRNA stability and does not work with other proteins to affect AREs-mediated *TNF* mRNA decay. Interestingly, Overexpressed MCPIP1 directly inhibited p65-induced promoter activation of *TNF* and *iNOS* in LPS treated cells (Liang et al., 2008). The block effect is specific as MCPIP1 can’t inhibit PPAR γ-induced PPREs promoter activation. Moreover, Huang et al. showed that MCPIP1 inhibited proinflammatory cytokines production by negatively regulated LPS-induced JNK signaling. Increased LPS-induced phosphorylated JNK was found in the lung of MCPIP1-deficient mice (Huang et al., 2013).

### At the post-transcriptional level

Cytokines production in an organism is strictly regulated starting from transcriptional initiation and ending with mature mRNA translation to a protein. The effect of post-transcriptional regulation is critical during this process.

CCCH zinc finger proteins can act as RNA-binding proteins to regulate cytokines transcripts metabolism (Fu and Blackshear, 2017). A massive number of studies have shown that MCPIP1 can directly target and degrade proinflammatory cytokines transcripts, acting as a negative regulator of inflammation. In contrast to tristetraprolin (TTP), a well-known member of CCCH zinc finger proteins that mediates mRNA decay by binding to AREs in the 3′UTRs of mRNAs, MCPIP1 binds and cleaves a specific stem-loop sequence in the 3′UTRs through its intrinsic endonuclease activity.

Let us take IL-6 as an example. IL-6 is a multifunctional proinflammatory cytokine, that plays an important role in various diseases. Many studies have shown that MCPIP1 can tightly regulate IL-6 production through its RNase activity (Matsushita et al., 2009). MCPIP1^−/−^ mice exhibited severe autoimmune disease, with significant splenomegaly and lymphadenopathy, and usually died within 12 weeks of birth. MCPIP1^−/−^ macrophages exhibited highly increased production of IL-6 and IL-12B under TLR ligand stimulation. PIN domain mutation abolished MCPIP1 RNase activity and prevented shortening of the *IL6* mRNA half-life. In adaptive immunity, MCPIP1 was shown to negatively regulated IL-17-mediated signaling by destabilizing *IL6* mRNA. Garg et al. found that the RNase activity of MCPIP1, but not its DUB function, is crucial in controlling the expression of various IL-17 induced genes (Garg et al., 2015). In addition to *IL6*, MCPIP1 degrades *IL17RA* and *IL17RC* mRNA and strongly inhibits the *LCN2* promoter activity by accelerating *NFKBIZ* mRNA decay (Monin et al., 2017). Interestingly, Mino et al. showed that MCPIP1 can collaborate with Roquin1, another CCCH zinc finger protein, to control IL-6 production (Mino et al., 2015). Both proteins recognize a common stem-loop structure but promote the *IL6* mRNA decay at different inflammatory response phases: MCPIP1 regulates the *IL6* mRNA decay at the early inflammatory phase by relying on the helicase activity of UPF1, mainly decreasing actively translated mRNAs, while Roquin 1 controls *IL6* mRNA decay at the later inflammatory phase by removing inactively translated mRNA.

Miao et al. compared inflammatory cytokines levels in the serum of MCPIP1^−/−^ and MCPIP1^+/+^ mice (Miao et al., 2013). Their results showed that the mRNAs of the T cell cytokines IL-17, IL-12, IL-4, IL-5 and TNFα were elevated in MCPIP1^−/−^ mice, but their upstream transcription factors, including T-bet, GATA3 and RORγ, were not changed. This result suggested that MCPIP1 directly regulates the metabolism of cytokines transcripts via a post-transcriptional process.

In addition to the 3′UTR of transcripts, MCPIP1 was reported to bind other mRNA regions. In work from Jiang et al., MCPIP1 was found to regulate *TET* mRNA by targeting its coding sequence (Jiang et al., 2016). MCPIP1 knockdown increased *TET* mRNA levels and then promoted the conversion of 5mC to 5hmC. Garg et al. also found that MCPIP1 targets the 5′ region of *IL17RA* mRNA (Garg et al., 2015). Although MCPIP1 possesses intrinsic RNase activity and can cleave diverse mRNAs without sequence preference, the mechanism by which it recognizes its substrates remains unknown.

### At the posttranslational level

MCPIP1 can act as a deubiquitinating enzyme to regulate TRAFs at the protein level. TRAFs are critical in the LPS-, IL-1B- and TNF- induced signaling pathway, including JNK and NF-κB activation (Muzio et al., 1998). As inappropriate regulation of the JNK and NF-κB pathways causes inflammation-induced tissue damage or malignancy, both signaling pathways should be tightly controlled to maintain transient activation, in which ubiquitination plays a key regulatory role. TRAFs are linked to K63 polyubiquitin chains early in the cellular response, which does not lead to protein degradation but is important for signal transduction (Deng et al., 2000).

MCPIP1 contains a unique DUB domain with no essential sequence similar to those in the five known DUB domains and possesses intrinsic deubiquitinase activity (Liang et al., 2010). Results have suggested that both PIN domain at the N-terminal region and the CCCH zinc finger domain are important for the deubiquitinatase activity of MCPIP1. The D141N, C157A, and C306R point mutations of MCPIP1 abolished its deubiquitinatase function, and the D141N mutation was also associated with its RNase activity. MCPIP1 could cleave both K48- and K63- linked polyubiquitin. Furthermore, MCPIP1 expression significantly decreased TRAF2 and TRAF6 ubiquitination and affects the K48-linked ubiquitination of IκBα.

In addition to its intrinsic deubiquitinatase activity, MCPIP1 was found to inhibit genotoxic NF-κB activation in a manner dependent on USP10, a member of the ubiquitin specific protease (USP) family (Niu et al., 2013). USP10 is recruited by MCPIP1 to cleave linear polyubiquitin chains from NF-κB essential modulator (NEMO), resulting in IKK activation and subsequent inhibition of NF-κB activation induced by DNA damage. Genotoxic treatment also induced the production of inflammatory cytokines through NF-κB signaling. Results showed that the mRNA levels of IL-6, TNFα and Cox-2 were substantially increased in MCPIP1^−/−^ cells. Consistently, USP10 knockdown rescued MCPIP1-mediated repression of DNA damage-induced inflammatory cytokine production. Their results showed that MCPIP1 can affect DNA damage signaling in cells after genotoxic treatment.

The deubiquitinatase activity of MCPIP1 contributes to macrophages polarization as well. Macrophages can be simply classified into 2 extreme types, M1 macrophages and M2 macrophages (Murray et al., 2014). When macrophages are stimulated by a variety of signals, such LPS or IL-1B, they are polarized to the M1 type and release proinflammatory cytokines, thus playing a protective role in the organism. In contrast, M2 macrophages are usually found in the tumor microenvironment, where they protect malignant cells through their immunosuppressive function (Gordon and Martinez, 2010). Kapoor et al. reported that the polarization of macrophages with DUB mutant and/or RNase mutant MCPIP1 to M2 type was much less effectively than the polarization of control macrophages. Loss of the deubiquitinatase activity of MCPIP1 inhibited IL-4 induced STAT6 and KLF4 activity and subsequently inhibit macrophages M2 polarization (Kapoor et al., 2015).

Importantly, MCPIP1 participates in the process of immune elimination of cancer cells. Death receptor 5 (DR5) is a cell surface marker that binds TNF-related apoptosis-inducing ligand (TRAIL). TRAIL is expressed by different immune cells, especially cytotoxic T cells. The binding of DR5 to TRAIL recruits Fas-associated death domain (FADD) to the cytoplasmic part of DR5 in cancer cells, after which FADD interacts with procaspase-8, forming the death-inducing signaling complex (DISC), which activates the caspase pathway to induce cell apoptosis. MCPIP1 inhibits the polyubiquitination of DR5 via its DUB activity and then enhance DR5 lysosomal degradation, resulting in resistance to DR5 activation or TRAIL-induced cancer cell apoptosis (Oh et al., 2018). MCPIP1 does not alter *DR5* mRNA levels but destabilizes DR5 protein through a posttranslational mechanism. The C157A mutation of MCPIP1 confirmed that DUB activity promotes autophagic/lysosomal degradation of DR5 and subsequently inhibits DISC formation.

### By inhibiting miRNA synthesis

Emerging studies have shown that miRNA expression is critical during the immune responses. Mice overexpressing miR-17-92 and miR-155 exhibited severe autoimmune and lymphoproliferative disease (Xiao et al., 2008; Costinean et al., 2009). MiR-155^−/−^ T cells exhibited increased expression of IL-4 (Rodriguez et al., 2007). MiRNA biogenesis was shown to be dynamically regulated by productive and abortive ribonucleases. MCPIP1 was reported to target the terminal loops of pre-miRNAs through its NYN nuclease domain, inhibiting miRNA synthesis (Suzuki et al., 2011). The ribonuclease function of MCPIP1 was found to compete with Dicer, a central ribonuclease in miRNA processing. MCPIP1 recognizes pre-miRNA through a vertebrate-specific oligomerization and preferentially cleaves the unpaired region of pre-miRNA near the terminal loop. Taking macrophages polarization as an example, TNFα upregulated miR155 expression and subsequently polarized macrophages to the M1 type. MCPIP1 suppressed the synthesis of miR155 and upregulated M2 polarization-associated miR223 and miR146 expression (Miao et al., 2013).

Nevertheless, whether MCPIP1 can regulate inflammatory cytokines mRNA by controlling miRNAs is controversial. Mino et al. reported that expression of the LPS-induced miRNAs such as miR155 and miR146 was not altered in mouse embryonic fibroblasts from MCPIP1^−/−^ mice and that miRNAs were not enriched by MCPIP1 CLIP-seq (Mino et al., 2015). In summary, further studies are needed to uncover whether miRNAs synthesis patriciates in MCPIP1-regulated cytokine production.

## HOW DO CYTOKINES REGULATE MCPIP1 EXPRESSION?

Various cells can express MCPIP1 and regulate their expression at different mechanistic levels, including the transcriptional, post-transcriptional and posttranslational levels. In the following section, we focus on how cytokines precisely inversely regulate MCPIP1 expression due to immune stimuli at different levels.

### At the transcriptional level

IL-1B interacts with IL-1R, initiating the adaptor protein myeloid differentiation primary response gene 88 (MyD88), which activates NF-κB signaling and subsequently upregulates the expression of *MCPIP1* (Skalniak et al., 2009). *MCPIP1* possesses four functional NF-κB binding sites within its second intron region and two NF-κB binding sites within its promoter sequence. NF-κB inhibitor κB (IκB) has been confirmed to inhibit IL-1B induced *MCPIP1* expression. In addition to NF-κB, Myd88 activates mitogen-activated protein kinase (MAPkinase) signaling. ERK MAPkinase pathway activation phosphorylates the transcription factor Elk-1, which binds to the promoter region of *MCPIP1*, initiating its transcription (Kasza et al., 2010). LPS, a potent inflammatory cytokines inducer, binds TLR4 and then induces the expression of *MCPIP1* through the same signaling pathway as IL-1B/IL1R (Huang et al., 2013). LPS/TLR4 recruits Myd88 and then activated NF-κB and ERK MAPkinase signaling as well. In addition to IL-1B and LPS binding, the binding of MCP-1 to its receptor, CCR2, induces Elk-1 phosphorylation and promotes *MCPIP1* transcription (O’Boyle et al., 2007). Serine/threonine-specific protein kinase (Akt) activation is also involved in MCP-1 induced *MCPIP1* transcription.

### At the post-transcriptional level and posttranslational level

At the post-transcriptional level, *MCPIP1* mRNA was found to be regulated by miR-9 in LPS-activated microglial cells (Yao et al., 2014). MiR-9 targets the *MCPIP1* 3′UTR and subsequently downregulates the expression of *MCPIP1*. Additionally, *MCPIP1* mRNA can be self-regulated by the MCPIP1 protein via its RNase activity (Yao et al., 2014). MCPIP1 targets the conserved stem-loop structure in the *MCPIP1* 3′UTR, similar to the *IL6* mRNA degradation process, inhibiting *MCPIP1* expression, and forming a negative feedback loop.

The mechanisms of MCPIP1 protein degradation in macrophages and T cells differ. Proteasomal degradation plays a key role in MCPIP1 degradation in macrophages (Iwasaki et al., 2011). Upon stimulation with TLR ligands, MCPIP1 is rapidly phosphorylated by IRAK1 and IκB kinase (IKK) β and then undergoes degradation via the UB-proteasome machinery. Both IRAK1 and IKK β are the downstream of MyD88, which suggests that when TLRs and IL-1R are stimulated by LPS or IL-1B, the activation of NF-κB signaling not only promotes *MCPIP1* transcription but also ready to degrade expressed MCPIP1 protein to maintain homeostasis. The mechanism of MCPIP1 protein degradation goes different in T cells. The protease Malt1 mediates MCPIP1 degradation via its linker region between PIN domain and N-terminal domain. Inactive C472A mutant *MALT1* failed to cleave MCPIP1 under TCR stimulation (Jeltsch et al., 2014).

## WHAT ROLE DOES THE MCPIP1/CYTOKINES NETWORK PLAY IN CANCER?

### Sustained angiogenesis, tissue invasion and metastasis

Limitless replication of cancer cells demands massive amounts of nutrition and oxygen. Angiogenesis, the formation of new blood vessels in the tumor microenvironment, guarantees a sufficient supply for growing malignant cells (Folkman, 2002). New vessels can be formed from circulating bone marrow-derived endothelial progenitor cells (BM-EPCs) or form directly from pre-existing vessels. Inflammatory is the main inducer of angiogenesis in cancer. Many studies have showed that proinflammatory cytokines, such as MCP-1, IL-1B, and IL-6, induce angiogenesis and promote tumor growth, invasion and metastasis (Voronov et al., 2003; Ono, 2008).

The effect of MCPIP1 in angiogenesis is controversial. Marona et al. reported that MCPIP1 expression is negatively correlated with clear cell renal cell carcinoma (ccRCC) progression and tumor vascularity (Marona et al., 2017). They found that MCPIP1 cleaves *IL8*, *VEGF* and *CXCL12* mRNA via its RNase activity, leading to the impaired chemotaxis of BM-EPCs, phosphorylation of VE-cadherin and vascular permeability. Mechanistic studies showed that decreased MCPIP1 in ccRCC is associated with increased *SDF-1* and *CXCR4* expression, which work together to promote tumor invasion and metastasis. However, Roy et al. showed MCPIP1 promotes angiogenesis in human umbilical vein endothelial cells (HUVECs) by inhibiting the production of antiangiogenetic miR-20b and miR-34a, which repress the translation of HIF-1α and SIRT-1 respectively (Roy et al., 2013). Niu et al. showed MCPIP1 activated the expression of angiogenesis-related gene *CDH12* and *CDH19* via its transcription factor activity in HUVECs, and then promoted vascularization (Niu et al., 2008). MCPIP1 may exhibit diverse actions according to normal or pathological conditions, but as limited amount of studies has been conducted, more evidences are needed to elucidate the behavior of MCPIP1 in cancer angiogenesis.

### Cancer cells apoptosis and replicative potential

Accumulating evidence has demonstrated that MCPIP1 acts as a potent tumor suppressor and induces cancer cells apoptosis. Studies in breast cancer showed that MCPIP1 directly binds and cleaves the mRNAs of multiple antiapoptotic genes, such as *BCL2A1*, *BCL2L1*, and *RELB*, and subsequently induces tumor cells apoptosis (Lu et al., 2016). In addition, miRNAs contribute to the proapoptotic activity of MCPIP1. Overexpression of MCPIP1 downregulated miR-3613-3p expression in neuroblastoma cells, which then upregulates apoptotic protease activating factor 1 (APAF1), causing cells apoptosis by caspase-9 proteolysis (Boratyn et al., 2016).

Limitless proliferation is another vital hallmark of cancer. In ccRCC cells, MCPIP1 depletion was found to significantly enhanced tumor cell viability and proliferation (Marona et al., 2017). MCPIP1 knockdown cells were observed to develop into larger tumor mass in NOD-SCID mice. Additionally, regulation of the microRNAs, including miR-155 and miR146a, by MCPIP1 was reported not only to be involved in immune responses, but also to participate in tumor proliferation (Suzuki et al., 2011).

### Impaired immune cells activation

Macrophages in the tumor microenvironment are usually polarized to the M2 type. M2 macrophages release anti-inflammatory cytokines, such as IL-10, promoting tumor cells proliferation and protecting malignant cells from chemotherapy induced apoptosis (Zheng et al., 2013; Murray, 2017; Singhal et al., 2019). Our previous work revealed that increased MCP-1 in the bone marrow in multiple myeloma induced MCPIP1 expression in macrophages. Overexpressed MCPIP1 polarized macrophages to the M2 type and promoted their ability to protect tumor cells via dual catalytic activities, resulting in drug resistance in myeloma cells (Xu et al., 2019).

As MCPIP1 is highly expressed in T cells, its function in T cells activation is of interest. A very large number of studies have shown that MCPIP1 negatively regulates T cells activation through various mechanisms, such as degradation of the immunoregulatory mRNAs *ICOS* and *REL* (Uehata et al., 2013). Moreover, MCPIP1 regulates DR5 expression through its deubiquitinase activity (Oh et al., 2018). DR5 expressed on the surface of cancer cells, can be recognized by TRAIL from cytotoxic T cells, inducing cancer cells apoptosis. Thus, downregulation of MCPIP1 in cancer cells decreases the effect of immune surveillance. Furthermore, the effect of MCPIP1 on the activation of other immune cells, such as B cells, natural killer cells and dendritic cells, remains to be determined.

## HOW DOES CLINICAL MANAGEMENT BENEFIT FROM THE MCPIP1/CYTOKINES NETWORK?

Although the interplay between immunity and cancer is complicated, researchers and clinicians are eager to translate novel, inspiring findings into practical, realistic management strategies.

Cytokines are the focus of immunity in cancer. There are simply two strategies of cytokines targeting in cancer management: nonselective inhibition and specific regulation. In nonselective strategies, systemic inflammation is broadly inhibited via unknown targets. Both non-steroidal anti-inflammatory drugs (NSAIDs) and steroid drugs have therapeutic potential in cancer treatment. Evidences has shown that NSAIDs can limit systemic inflammation and improve cachexia. In several clinical studies of colorectal cancer, NSAIDs used as adjuvant therapy and presented promising benefits (Gierach et al., 2008; Algra and Rothwell, 2012; Rothwell et al., 2012). Steroid drugs such as dexamethasone are more practical than NSAIDs in cancer treatment (Leggas et al., 2009). For example, dexamethasone is an indispensable part of almost every chemotherapy regimen in the treatment of multiple myeloma. The use of dexamethasone improves the efficiency of chemotherapy and decreases drugs-associated toxic effects (Garderet et al., 2018).

For the selective targeting of specific cytokines, although multiple early-phase clinical trials are being conducted, there are few ideal drugs have been found to be effective in clinical practice, as observed in research studies. For instance, Carlumab, an MCP-1 specific monoclonal antibody, can directly against the binding of MCP-1 to its receptor, inhibiting downstream signaling activation and overcoming MCP-1 promoted tumor progression (Brana et al., 2015). Carlumab was proven to have promising beneficial antitumor properties in preclinical studies, but a phase 1b clinical study showed that it could not sustainably inhibit the serum MCP-1 concentration in patients; thus, further clinical use of Carlumab is not recommended. The complexity of crosstalk and compensation within the tumor microenvironment might explain the failure of specific cytokines-targeted drugs in cancer treatment.

This failure is why we switch our focus to the downstream regulator MCPIP1. Multiple studies have shown that MCPIP1 is important during the tumorigenesis, including that in neuroblastoma, ccRCC and breast cancer cells (Boratyn et al., 2016; Lu et al., 2016; Marona et al., 2017). In ccRCC, the MCPIP1 expression is negatively correlated with tumor grade. The same negative correlation was observed in breast cancer, and decreased MCPIP1 expression is associated with enhanced metastatic characteristics. Considering the expression profile of MCPIP1 in tumor progression, it has the potential to act as a potent prognosis marker in cancers. However, further studies in more cancer types are needed to uncover the predictive value of MCPIP1.

Moreover, a growing number of studies contributed to interfere the effect of MCPIP1 to explore its role in cancer treatment. It has been reported that MG-132, a proteasome inhibitor, effectively upregulates MCPIP1 expression, potently activating the apoptosis of cancer cells (Skalniak et al., 2013). However, as MCPIP1 acts as an immumosuppressor in cancer-related immunity, MCPIP1 expression has extremely different effects in immune cells and cancer cells. Cancer cells benefit from downregulated MCPIP1, which is related to increased antiapoptotic gene mRNA expression and decreased expression of DR5 on the cancer cell surface. All these effects protect cancer cells from death. In contrast, downregulated MCPIP1 polarizes macrophages to the M1 type and promotes immune activation of T cells. Specific downregulation of MCPIP1 expression can enhance the antitumor effect of immune cells. Therefore, determining how to specifically interfere with MCPIP1 expression in cancer cells or immune cells is a major challenge for translating research findings to clinical practice.

## CONCLUSION AND PERSPECTIVES

Cytokines have extremely vital and complicated biological functions, and their abnormal expression is involved in many pathological processes, including autoimmune disease and cancers. Various cell types and multiple mechanisms participate in the tight regulation of cytokines production. MCPIP1, which possesses a unique deubiquitination domain and has intrinsic RNase activity, was reported to effectively inhibit immune activation. However, the role of MCPIP1 in cancer has not drawn sufficient attention to date and only a few studies on this topic have been published.

MCPIP1 is at the center of cytokines regulatory networks. It mutually regulates cytokines at several levels, ranging from transcriptional to post-transcriptional and posttranslational processes. Moreover, specific cytokines can regulate other cytokines and MCPIP1 also has a negative feedback effect on itself. Cytokines and MCPIP1 work together to form a complicated network to precisely and timely regulate intracellular responses in the cancer environment. It is well established that cytokines expression associated with tumor progression and that some specific cytokines can act as prognostic markers. However, very few direct cytokines-targeting drugs have shown promising effects in clinical trials. And there are also no MCPIP1-targeting drugs available yet in pre-clinical or clinical studies. Thus, further studies should be conducted to determine whether interference with the crosstalk between cytokines and MCPIP1 has potential benefits in cancer treatment, and more studies are needed to elucidate the role of MCPIP in cancer.
